# Bicuspid aortic valve formation: *Nos3* mutation leads to abnormal lineage patterning of neural crest cells and the second heart field

**DOI:** 10.1242/dmm.034637

**Published:** 2018-10-19

**Authors:** Joshua C. Peterson, Mary Chughtai, Lambertus J. Wisse, Adriana C. Gittenberger-de Groot, Qingping Feng, Marie-José T. H. Goumans, J. Conny VanMunsteren, Monique R. M. Jongbloed, Marco C. DeRuiter

**Affiliations:** 1 Dept. Anatomy and Embryology, Leiden University Medical Center, PO Box 9600, 2300 RC Leiden, The Netherlands; 2 Dept. Cardiology, Leiden University Medical Center, PO Box 9600, 2300 RC Leiden, The Netherlands; 3 Dept. Physiology and Pharmacology, Schulich Medicine & Dentistry, Western University, London, ON N6A 5C1, Canada; 4 Dept. Molecular Cell Biology, Leiden University Medical Center, PO Box 9600, 2300 RC Leiden, The Netherlands

**Keywords:** Nos3, Bicuspid aortic valve, Lineage tracing, Embryo, Development, Outflow tract

## Abstract

The bicuspid aortic valve (BAV), a valve with two instead of three aortic leaflets, belongs to the most prevalent congenital heart diseases in the world, occurring in 0.5-2% of the general population. We aimed to understand how changes in early cellular contributions result in BAV formation and impact cardiovascular outflow tract development. Detailed 3D reconstructions, immunohistochemistry and morphometrics determined that, during valvulogenesis, the non-coronary leaflet separates from the parietal outflow tract cushion instead of originating from an intercalated cushion. *Nos3^−/−^* mice develop a BAV without a raphe as a result of incomplete separation of the parietal outflow tract cushion into the right and non-coronary leaflet. Genetic lineage tracing of endothelial, second heart field and neural crest cells revealed altered deposition of neural crest cells and second heart field cells within the parietal outflow tract cushion of *Nos3^−/−^* embryos. The abnormal cell lineage distributions also affected the positioning of the aortic and pulmonary valves at the orifice level. The results demonstrate that the development of the right and non-coronary leaflets are closely related. A small deviation in the distribution of neural crest and second heart field populations affects normal valve formation and results in the predominant right-non-type BAV in *Nos3^−/−^* mice.

## Abstract (precis)

**Summary:** The endothelial *Nos3* mutation impacts neural-crest and second-heart-field lineage patterning within the aortic valve, which affects outflow-tract formation, as well as aortopulmonary angulation.

## INTRODUCTION

The tricuspid aortic valve (TAV) has a crucial role in maintaining unidirectional blood flow from the left ventricle into the systemic circulation. Abnormalities in aortic valve morphology, as seen in the case of the bicuspid aortic valve (BAV), have been linked to valvular regurgitation, stenosis and progressive thoracic aortic aneurysm development (Ward, 2000; Verma and Siu, 2014; Sievers et al., 2015; Merkx et al., 2017). A BAV is currently accepted as a congenital anomaly with a high incidence, occurring in 0.5-2% of the Western population (Roberts, 1970; Ward, 2000; Hoffman and Kaplan, 2002).

BAV formation is generally considered to be an abnormal fusion of aortic leaflets occurring during embryonic development (Fernández et al., 2009; Théron et al., 2015; Odelin et al., 2017). Although leaflet fusion might be a valid mechanistic explanation, there are currently no unequivocal data supporting this mechanism in BAV mouse models.

During embryonic development the heart starts as a single heart tube consisting of an outer layer of myocardium and an inner lining of endocardium. These layers are separated by a thick hydrophilic region of cardiac jelly, rich in extracellular matrix, hyaluronic acid, glycosaminoglycans and proteoglycans, produced by cardiomyocytes (Manasek, 1968). The primitive myocardium secretes factors, such as Bmp2, in the cardiac jelly that induce the transition of endothelial cells into mesenchymal cells (EMT) (Sugi et al., 2004). This process results in an invasion of endothelial-derived mesenchymal cells into the cardiac jelly (Eisenberg and Markwald, 1995; Kisanuki et al., 2001). In the cardiac outflow tract (OFT), EMT results in the formation of a septal and a parietal cushion, the primordia of the myocardial OFT septum and the semilunar valves. Defects in cardiac jelly synthesis result in severely hypoplastic cushions due to failed EMT (Baldwinet al., 1994; Camenisch et al., 2000). Failure of EMT has been shown to result in BAVs (Thomas et al., 2012; Mommersteeg et al., 2015). BAV formation in *Nos3^−/−^* has also been suggested to be caused by early defects in EMT resulting in reduced mesenchyme populations in the OFT cushions (Fernández et al., 2009; Liu et al., 2013).

**Fig. 1.**
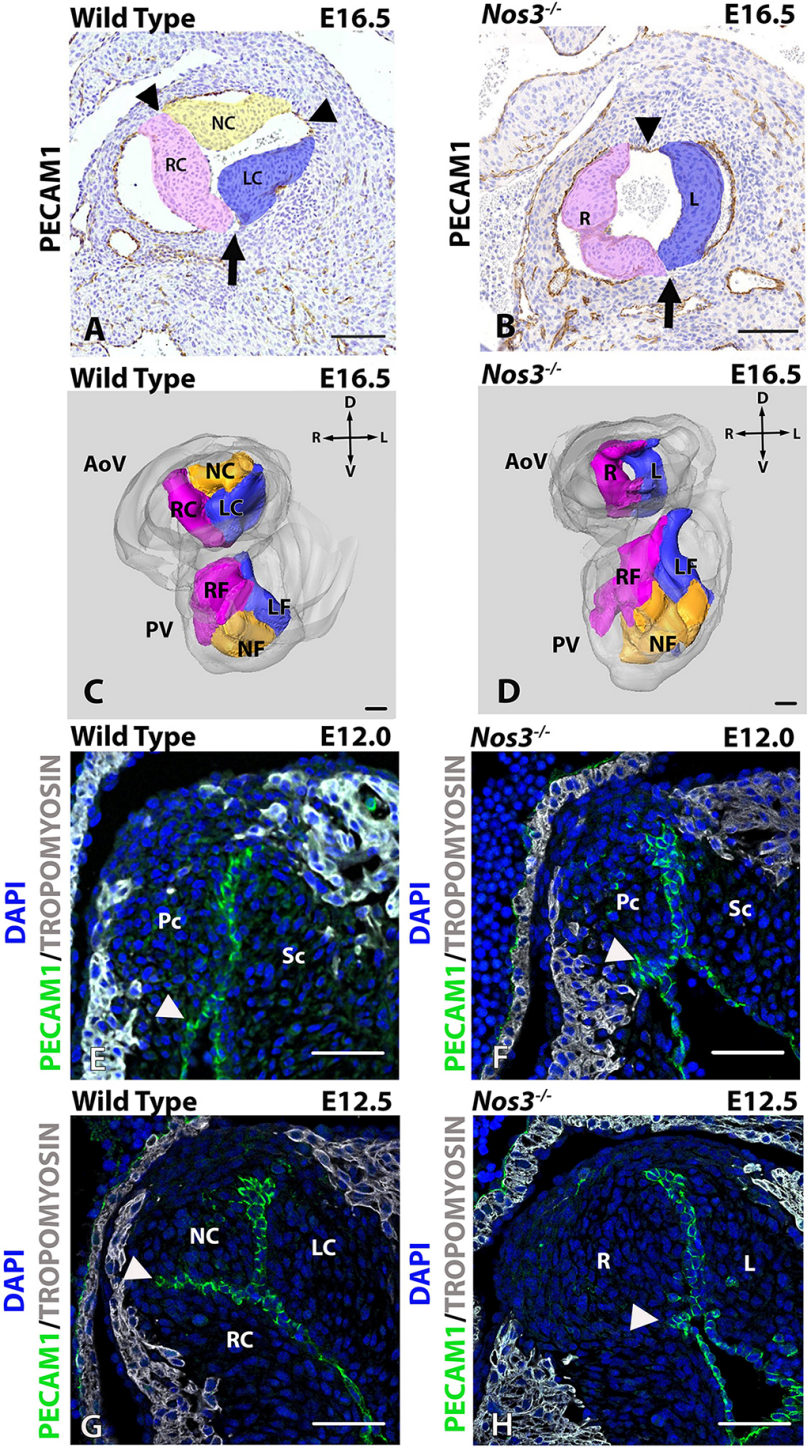
**Failure of cushion separation results in bicuspid aortic valves (BAVs).** (A,B) Anti-PECAM1-labelled histological antibody staining depicting the left coronary leaflet (LC), right coronary leaflet (RC) and non-coronary leaflet (NC) in E16.5 tricuspid aortic valve (TAV) wild-type (A), and left (L) right (R) leaflets in BAV *Nos3^−/−^* (B) mice. Position of the facing L-R commissure was similar between wild-type and *Nos3^−/−^* mice (indicated by arrows in A and B). BAV *Nos3^−/−^* mice developed a commissure opposite to the facing commissure, whereas TAV wild-type mice developed three commissures equilateral between the leaflets (arrowheads in A and B). (C,D) 3D reconstruction of the aortic and pulmonary valves (AoV and PV, respectively) showing individual and connected leaflets within the aortic root in wild-type (C) and *Nos3^−/−^* (D) mice. Note that, in *Nos3^−/−^* mice, leaflets of the PV developed normally. (E-H) Anti-PECAM1 (green) and anti-tropomyosin (TM; grey) immunofluorescently stained paraffin sections of the aortic valve in E12.0 wild-type (E) and *Nos3*^−/−^ (F) embryos show the parietal (Pc) and septal (Sc) cushion. Wild-type embryos develop tricuspid leaflets as a result of separation of the parietal cushion into the right- and non-coronary leaflets at E12.5 (G). Failure of separation of the parietal cushion resulted in the formation of BAVs (H). Location of endothelial infolding is indicated by white arrowheads. The endothelial distribution within the leaflets showed no indication of leaflet fusion as the result of the merging of two individual leaflets, as no endothelial-lined raphe is present in bicuspid *Nos3*^−/−^ mice. Nuclei were stained with DAPI (blue). AoV, aortic valve; PV, pulmonary valve; Pc, parietal cushion; Sc, septal cushion; RC, right coronary; LC, left coronary; NC, non-coronary; RF, right facing; LF, left facing; NF, non-facing; R, right; L, left; V, ventral; D, dorsal. Scale bars: 50 µm.

Migration of cardiac neural crest cells from the neuroectoderm into the OFT cushions induces the formation of the aortopulmonary (AP) septum, which divides the common OFT at the cardiac-to-vascular border into an aortic and pulmonary orifice, and more proximally located intracardiac tissue into a right and left ventricular OFT (Waldo et al., 1998; Jiang et al., 2000; Gittenberger-De Groot et al., 2005). During further development, the parietal cushion gives rise to, at the orifice level, the right-facing leaflets of the aortic and the pulmonary valve, while the septal cushion will develop into the left-facing leaflets of both valves. Finally, the non-facing aortic leaflet and pulmonary leaflet are considered to be derived from separately developing intercalated cushions on the posterior and anterior sides of the OFT, respectively (Kramer, 1942; Lin et al., 2012). Although the development of the septal and parietal cushion has been studied intensively, the role of these intercalated cushions during development remains a challenging concept despite recent progress (Anderson et al., 2003; Lin et al., 2012; Eley et al., 2018; Mifflin et al., 2018). For clarity of description of the valve leaflets and the correlation with the terminology used for the aortic leaflets in human patients with BAV, we will refer to the aortic leaflets as right coronary (RC), left coronary (LC) and non-coronary (NC) leaflets (Sievers and Schmidtke, 2007). For the pulmonary semilunar valve leaflets we have chosen to use right-facing (RF), left-facing (LF) and a non-facing (NF) leaflets (Fig. 1A-D).

It is well established that the aorta and aortic valve are developmentally related. Neural crest (Waldo et al., 1998; Jiang et al., 2000), endothelial (Eisenberg and Markwald, 1995; Kisanuki et al., 2001), epicardial cell lineages (Gittenberger-de Groot et al., 2012) and second heart field (SHF) (Zaffran and Kelly, 2012)-derived cells contribute to both the ascending aorta, the aortic valve (Eley et al., 2018; Mifflin et al., 2018) and the various components of the aortic root (valvular leaflets, annulus, sinuses of Valsalva) (de la Cruz et al., 1977; Kirby et al., 1983; Waldo et al., 2001). Mouse models of BAVs show cushion formation to be an essential process during valve formation, and defects in the contributing cell lineages are known to result in BAVs (Biben et al., 2000; Lee et al., 2000; Macatee, 2003; Laforest and Nemer, 2011; Makki and Capecchi, 2012; Thomas et al., 2012; Mommersteeg et al., 2015). In the present study, we examined the contributions of neural crest, endothelial and SHF lineages in aortic valve development of wild-type and *Nos3^−/−^* mouse embryos to identify novel congenital aberrations involved in the formation of a BAV. Understanding the fundamental embryology of these early cardiac lineages is crucial to address the challenges in BAV pathology.

## RESULTS

### Morphological landmarks in bicuspid *Nos3*^−/−^ mice

Seventy-three percent of *Nos3^−/−^* embryos had a normal TAV, while 27% develop a BAV (Table S4). In *Nos3*^−/−^ embryos, the BAV had two similarly sized leaflets in a left-right leaflet orientation without a visible raphe that indicated the position of a possible third commissure (Fig. 1A,B). The lack of a raphe did not allow for discrimination between an R-N (a fusion/confluence of RC and NC leaflets)- or L-N (a fusion/confluence of LC and NC leaflets)-type BAV solely on the basis of morphological aspects. In wild-type mice, the three parabolic-shaped leaflets are embedded within the aortic root. Distally, at the sinotubular junction, these structures formed three interleaflet commissures. In contrast to wild type, bicuspid *Nos3^−/−^* mice developed a commissure (arrowheads, Fig. 1A,B) opposite to the facing commissure (arrow, Fig. 1A,B).

### Incomplete separation of the parietal cushion leads to an R-N BAV in *Nos3*^−/−^ embryos

The first indications of leaflet formation could be observed at E12.0, at which point the putative aortic valve leaflets consisted of the parietal and septal cushions and a slight indentation of endothelial cells was present in both wild-type and *Nos3^−/−^* embryos (Fig. 1E,F, arrowheads). At E12.5, a marked separation of the parietal cushion was observed in wild-type embryos. The RC and NC leaflet could be distinguished by the presence of an endothelial infolding into the cushion (Fig. 1G, arrowhead). Bicuspid *Nos3^−/−^* embryos did not develop this marked endothelial infolding, causing an incomplete separation of the parietal cushion into an NC and RC leaflet (Fig. 1H). This resulted in the formation of a single right valvular leaflet, leading to an R-N BAV. Moreover, there was no indication of intercalated cushion development in wild-type embryos between the parietal and septal cushion between E10.5 and E12.5 (Fig. S1).

### Aortic leaflets develop solely from the parietal and septal cushions

**Fig. 2.**
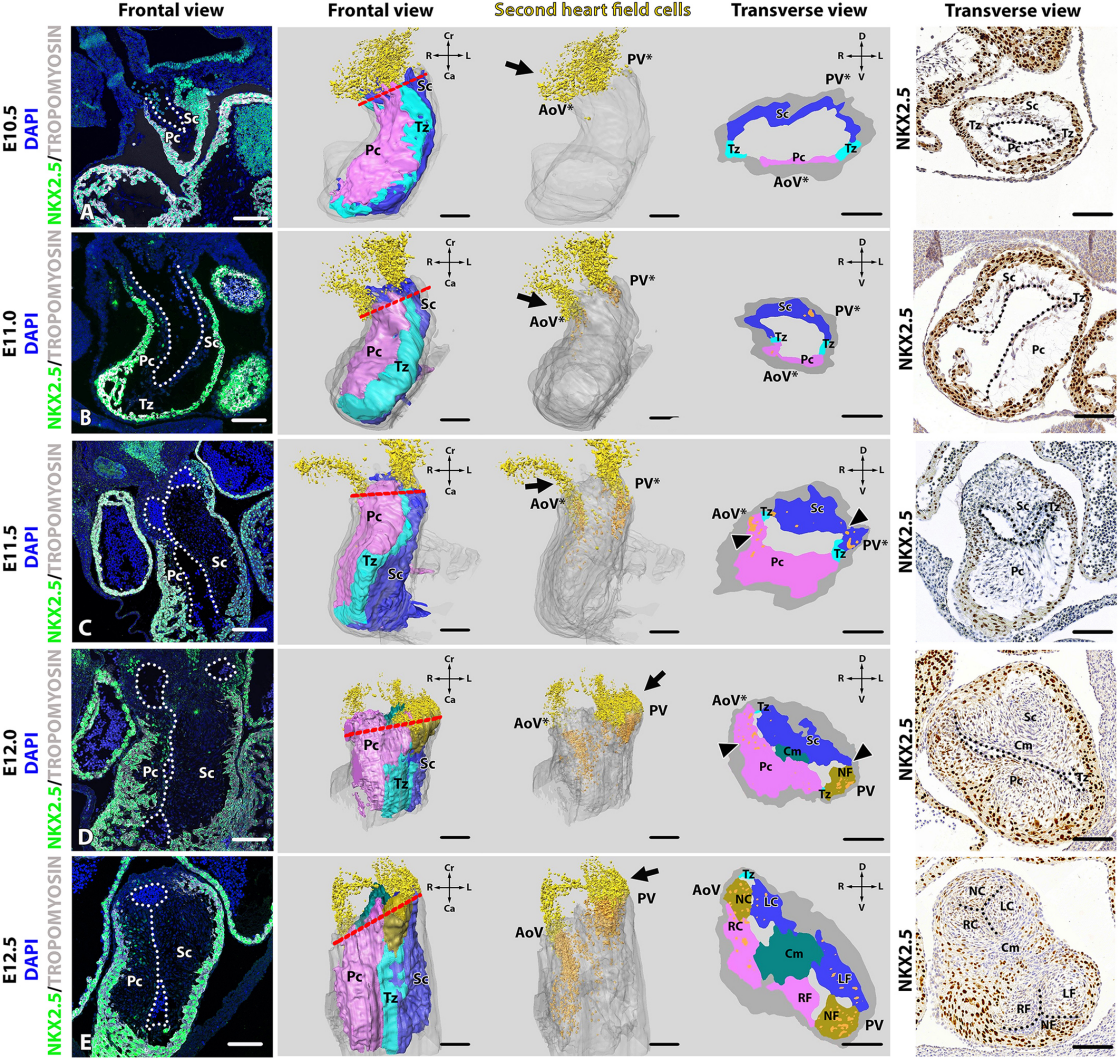
**Aortic and pulmonary leaflets develop from the parietal and septal cushions.** The first column depicts frontal fluorescent images of embryonic septal (Sc) and parietal (Pc) cushions within the cardiac outflow tract (OFT) of wild-type embryos aged E10.5-E12.5 using anti-NKX2.5 (green), anti-tropomyosin (TM; grey) and DAPI as a nuclear marker (blue). Columns 2 and 4 show a frontal view of and transverse sections through 3D reconstructions of the OFT that can be divided into the myocardial outer wall (grey), and the Pc and Sc (purple and dark blue, respectively). The parietal and septal cushions were connected by a thin transitional zone (Tz) of cardiac jelly sparsely populated by cells (cyan). Column 3 is similar to the reconstructions in column 2 but without the reconstructed Pc, Tz and Sc to demonstrate the intracardiac SHF cells. The 5th column depicts an immunostaining of NKX2.5 at the location of the transverse plane (red dotted line). (A) NKX2.5^+^/TM^−^ second heart field (SHF) cells (yellow) were located at the proximal OFT outside the heart tube at E10.5 (arrows). (B) NKX2.5^+^/TM^−^ SHF cells that were positioned in the septal and parietal cushions at opposing poles of the OFT at E11.0 have been reconstructed in orange. (C) Due to OFT rotation, the NKX2.5^+^ cells (orange) could be found within the parietal and septal cushions where the non-facing (NF) and non-coronary (NC) leaflets would form (arrowheads). (D) At E12.0, condensed mesenchyme (Cm; dark green) indicated the formation of the aortopulmonary (AP)-septum in the transverse view. At the pulmonary orifice, the NF leaflet dissociated from the septal cushion whilst the NC leaflet remains connected (arrowheads). (E) Complete separation of valve leaflets was established at E12.5, at which point the NC leaflet dissociated from the parietal cushion. AoV*, future aortic valve; PV*, future pulmonary valve; AoV, aortic valve; PV, pulmonary valve; Pc, parietal cushion; Sc, septal cushion; Tz, transitional zone; Cm, condensed mesenchyme; RC, right coronary leaflet; LC, left coronary leaflet; NC, non-coronary leaflet; RF, right-facing leaflet; LF, left-facing leaflet; NF, non-facing leaflet; D, dorsal; V, ventral; Cr, cranial; Ca, caudal. White and black dotted lines indicate endothelial lining. Scale bars: 100 µm.

The early OFT cushion formation started around E10.5 in wild-type mice with the production of cardiac jelly within the interstitial space between the outer myocardial wall and inner endocardial lining, followed by migration of endocardial-derived cells into two compartments, resulting in the two OFT cushions: the parietal and septal cushions (Fig. S1). During cellularization the two cushions remained interconnected by two thin transitional zones. The transitional zones are two distinct regions which connect the parietal and septal cushion over the complete length at both ends and are characterized by their composition of mostly cardiac jelly, virtually devoid of cells (Fig. 2A, Fig. S1). At E10.5 and E11.0, the parietal cushion was positioned ventrolaterally in the cardiac OFT, whereas the septal cushion was located more dorsomedially (Fig. 2A,B). Extracardiac SHF cells could be observed immunohistochemically as non-myocardial [tropomyosin (TM) negative] cells expressing nuclear NKX2.5 (Waldo et al., 2001) (Fig. S2). NKX2.5^+^/TM^−^ SHF cells (Fig. 2, Fig. S3; yellow) migrated via the pharyngeal arteries into the distal parts of the parietal and septal cushions into the cardiac OFT (Fig. 2, Fig. S3; orange). From E11.5 onwards, the future pulmonary OFT changed from a left medial position to a more ventral position with regard to the future aortic ostium, corresponding to a clockwise rotation (Fig. 2C-E, Fig. S3C-E). This phenomenon has been described as resulting from the asymmetric addition of SHF cells to the pulmonary side of the OFT, the so-called ‘pulmonary push’ (Scherptong et al., 2012). The parietal cushion became positioned more dorsal and the septal cushion more ventral (Fig. 2D,E). At this stage, the NKX2.5^+^/TM^−^ SHF cells form a distinct group of cells in the septal and parietal cushions close to the transitional zones (Fig. 2D,E; cyan, Fig. S3D,E). The transitional zones in between the septal and parietal cushions remained sparsely populated by cells. The interleaflet commissures of the LC and NC aortic leaflets as well as the LF and NF pulmonary leaflets developed at these transitional zones. The pulmonary NF leaflet also derived from a main OFT cushion (in this case, the septal cushion). The timing is somewhat different in that this occurred at E12.0 (Fig. 2D), whilst the separation of the aortic NC leaflet from the parietal cushion occurred somewhat later, at E12.5 (Fig. 2E). The septal cushion therefore gave rise to three leaflets: the LC aortic leaflet as well as the LF and NF pulmonary leaflets. The parietal cushion also gave rise to three leaflets: the RC and NC aortic leaflets as well as the RF pulmonary leaflet (Fig. 2E).

### The volume of the myocardium and the OFT cushions are not affected in *Nos3*^−/−^ embryos

Developmental growth of the OFT cushions could be determined by a decrease in acellular fraction volume (as a measure for the amount of cardiac jelly) and an increase in cellular fraction volume containing cell bodies. Histological image examination determined no difference (*P*>0.05) in cushion composition of the septal and parietal cushions between *Nos3^−/−^* and wild-type embryos from stages E10.5-E12.5 (Fig. S4A-B). These findings indicate that defects in *Nos3^−/−^* embryos were not caused due to a reduction in total cell volume or changes in cardiac jelly production. Furthermore, no differences were found in heart size based on ventricular myocardial volume measurements between wild type and *Nos3^−/−^* embryos at embryonic age E10.5-E13.5 indicating that the myocardial growth rate is unaffected by the *Nos3* mutation (Fig. S4C).

### Aortic and pulmonary leaflets harbour unique cell lineage distributions

The use of the Cre-*loxP* system allowed the study of cellular offspring by genetically labelling original progenitor cells (Sauer and Henderson, 1988; Nagy, 2000). By combining genetic lineage tracing and antibody staining it was confirmed that the NKX2.5^+^/TM^−^ population within the NC leaflets of the aorta are of SHF descent and that these do not colocalize with endothelial (Fig. S5A) or neural-crest  (Fig. S5B)-derived populations, but are a subpopulation of the *Mef2c*-derived SHF cells (Fig. S5C). These NKX2.5^+^/TM^−^ SHF populations are also found in the NF leaflet in the pulmonary orifice (Fig. S5D-F). Recently, studies by Mifflin et al. and Eley et al. have identified similar populations in the NC leaflet of the aorta (Eley et al., 2018; Mifflin et al., 2018).

**Fig. 3.**
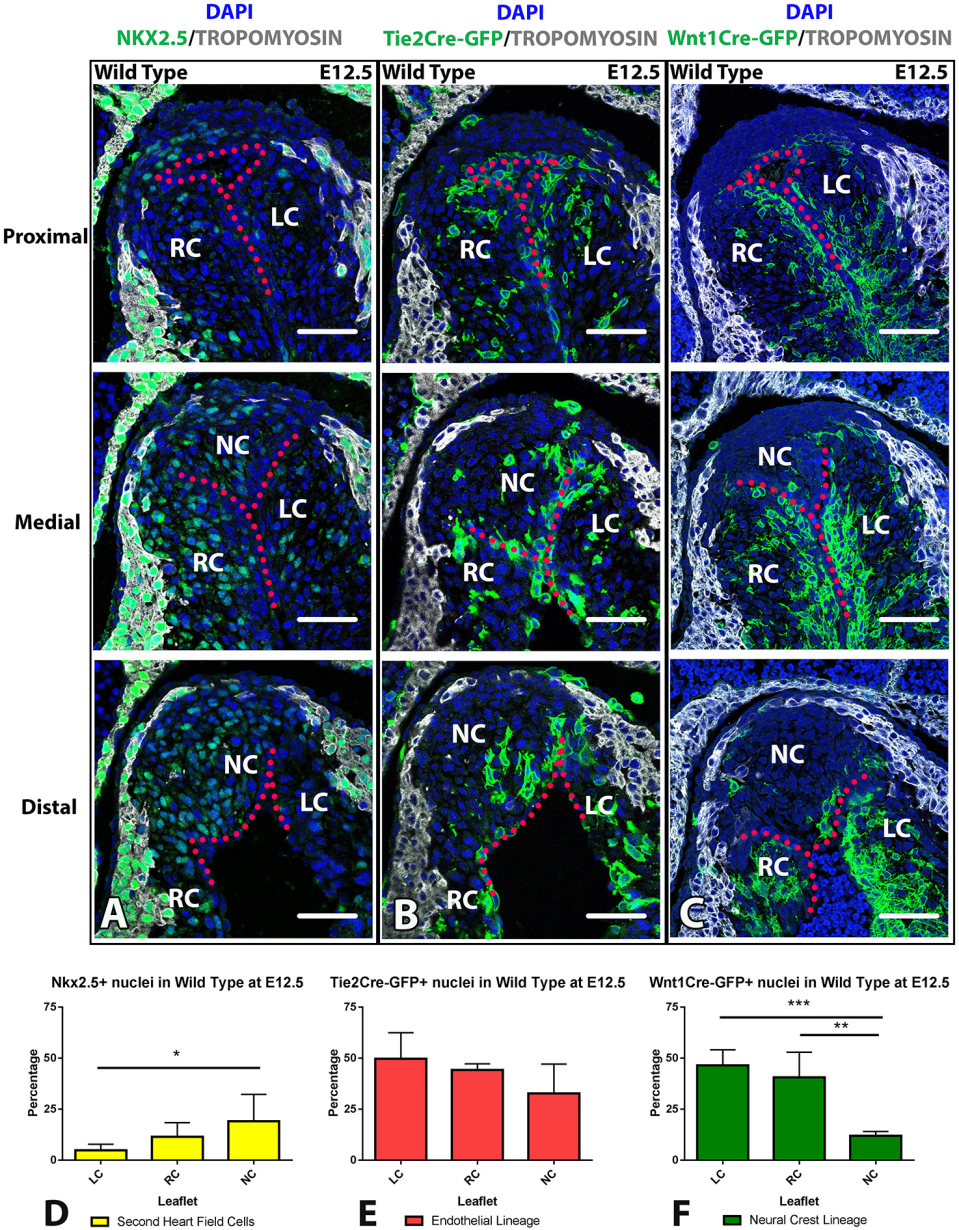
**Aortic valve leaflets harbour unique cell lineage distributions in wild-type embryos at E12.5.** (A) Top to bottom, pictures represent NKX2.5^+^/TM^−^ second heart field (SHF; green) cell distribution from proximal to distal regions within the aortic valve. Note that the SHF populations within the valve are localized at medial and distal positions. (B) Depiction of the *Tie2Cre*-GFP^+^ endothelial lineage (green) contribution throughout the aortic valve. (C) *Wnt1Cre*-GFP^+^ neural crest lineage (green) cell contribution to the aortic valve is primarily organized in the right and left coronary (RC and LC) leaflets but not the non-coronary (NC) leaflet. (D) Cell lineage leaflet analysis of NKX2.5^+^/TM^−^ SHF showed significantly more contribution to the NC leaflet than to the LC leaflet of the aortic valve in wild-type embryos (*n*=8) at E12.5 (*P*<0.05). (E) *Tie2Cre–*GFP^+^ endothelial-derived cells showed no bias as to which leaflet is populated, and distributed equally among individual leaflets in wild-type embryos (*n*=5) at E12.5. (F) In wild-type embryos, *Wnt1Cre*-GFP*^+^* neural-crest-derived cells contributed more to the RC (*P*<0.01) and LC (*P*<0.001) leaflets and less to the NC leaflet at E12.5 (*n*=4). Data are mean±s.d. *P<0.05, **P<0.01 and ***P<0.001, determined by one-way ANOVA. Colour scheme: DAPI (blue), tropomyosin (TM; grey), lineage markers (green), endothelial lining represented with red dotted line. RC, right coronary leaflet; LC, left coronary leaflet; NC, non-coronary leaflet. Scale bars: 50 µm.

Cell lineage image quantification determined specific regions in the aortic valve to which early cardiac cell lineages contributed. In wild-type embryos, the majority of NKX2.5^+^/TM^−^ SHF cells contributed to the NC leaflet (Fig. 3A,D), whereas cells of neural crest origin primarily contributed to the LC and RC leaflets (Fig. 3C,F). Similar neural crest patterns have also been reported in other studies (Phillips et al., 2013; Odelin et al., 2017). Interestingly, endothelial-derived cells were equally distributed among the aortic leaflets in wild-type embryos (Fig. 3B,E).

### The distribution of the SHF and neural crest cells in the cardiac OFT is affected in *Nos3*^−/−^ embryos

**Fig. 4.**
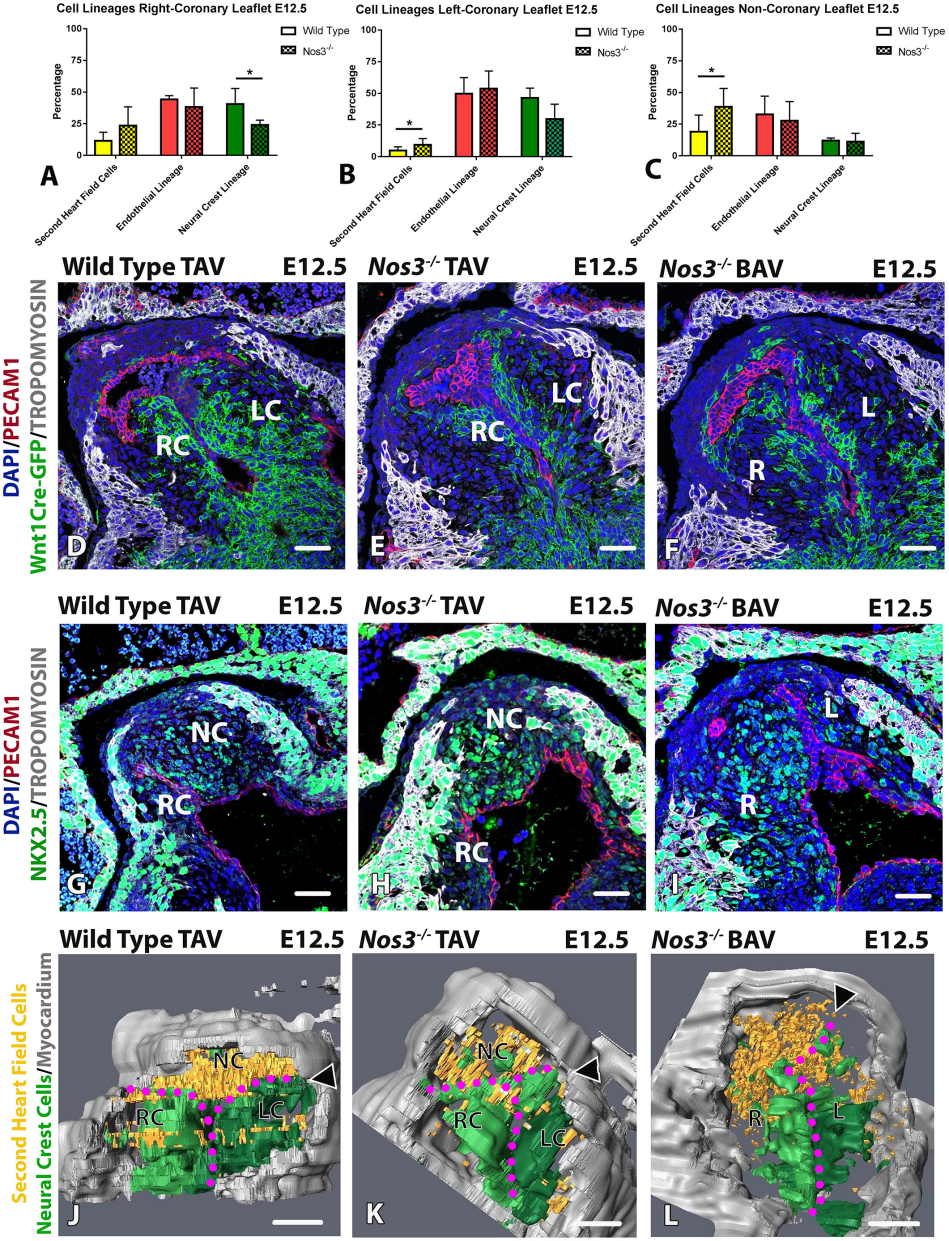
**Aberrant neural crest and SHF lineage distribution in *Nos3*^−/−^ embryos.** (A) Cell lineage analysis in *Nos3*^−/−^ embryos showed that significantly fewer neural-crest-derived cells contribute to the right coronary (RC) leaflet in *Nos3*^−/−^ than in wild-type embryos at E12.5 (*P<0.05). (B) Increased contribution of NKX2.5^+^/TM^−^ SHF cells was observed in the left coronary (LC) leaflet in *Nos3^−/−^* embryos as compared to age-matched wild-type embryos (*P<0.05). (C) The NC leaflet of *Nos3*^−/−^ contains a significantly larger NKX2.5^+^/TM^−^ SHF population than those found in wild-type embryos at E12.5 (*P*<0.05). (D-F) Immunofluorescent images of *Wnt1-Cre;mTmG* tricuspid aortic valve (TAV) wild-type (D), *Nos3*^−/−^ TAV (E) and bicuspid aortic valve (BAV) *Nos3*^−/−^ (F) embryos at E12.5 showed diminished neural crest populations (green) in the leaflets of the aortic valve in *Nos3^−/−^* embryos. (G-I) Immunofluorescent images of wild-type TAV (G), *Nos3^−/−^* TAV (H) and *Nos3^−/−^* BAV (I) show increased NKX2.5^+^/TM^−^ SHF cells in the aortic leaflets of *Nos3^−/−^* embryos. Colour scheme: anti-PECAM1 (red), anti-tropomyosin (TM; grey), lineage marker (green), nuclei were stained with DAPI (blue). (J-L) 3D reconstructions showed that the NKX2.5^+^/TM^−^ SHF cells (orange) primarily localize in the NC leaflet in wild-type TAV and *Nos3*^−/−^ TAV mice, whereas, in *Nos3*^−/−^ BAV mice, the majority of SHF cells were located in the right (R) leaflet. BAV embryos developed the position of the left commissure (arrowheads) more posteriorly than wild-type TAV and *Nos3*^−/−^ TAV mice. Data are mean±s.d. for *n*≥4 mice per group. *P<0.05 determined by two-tailed Student's *t*-test. RC, right coronary leaflet; LC, left coronary leaflet; NC, non-coronary leaflet. Scale bars: 50 µm.

The total leaflet volume between wild-type and *Nos3*^−/−^ embryos did not differ (Fig. S6). *Nos3^−/−^* embryos had a significantly reduced population of neural-crest-derived cells in the RC leaflet when compared to age-matched wild-type embryos (Fig. 4A). Moreover, a larger contribution of NKX2.5^+^/TM^−^ SHF cells was observed in the LC (Fig. 4B) and NC (Fig. 4C) leaflet in *Nos3^−/−^* embryos. Although all *Nos3^−/−^* embryos had defects in neural crest and SHF populations, only 27% of *Nos3^−/−^* embryos develop a BAV, as reported in earlier studies of *Nos3*^−/−^ mice (Table S4 and Fernández et al., 2009).

These results show that an altered distribution of neural crest and SHF populations is present in the developing abnormal leaflets of BAV (Fig. 4D-I). Three-dimensional reconstruction of the neural crest and SHF populations within the leaflets showed that these regional lineage disturbances were accompanied by rotational anomalies within the annulus, impacting commissure positioning in *Nos3^−/−^* embryos (Fig. 4J-L). As a result, the left commissure developed more dorsally in *Nos3^−/−^* BAV when compared to wild-type TAV and *Nos3^−/−^* TAV embryos (Fig. 4J-L). Measurement of extracardial SHF and neural crest populations showed an equal volume outside the heart, which indicates that these cell populations are most probably not affected prior to their homing into the cardiac cushions (Fig. S7).

**Fig. 5.**
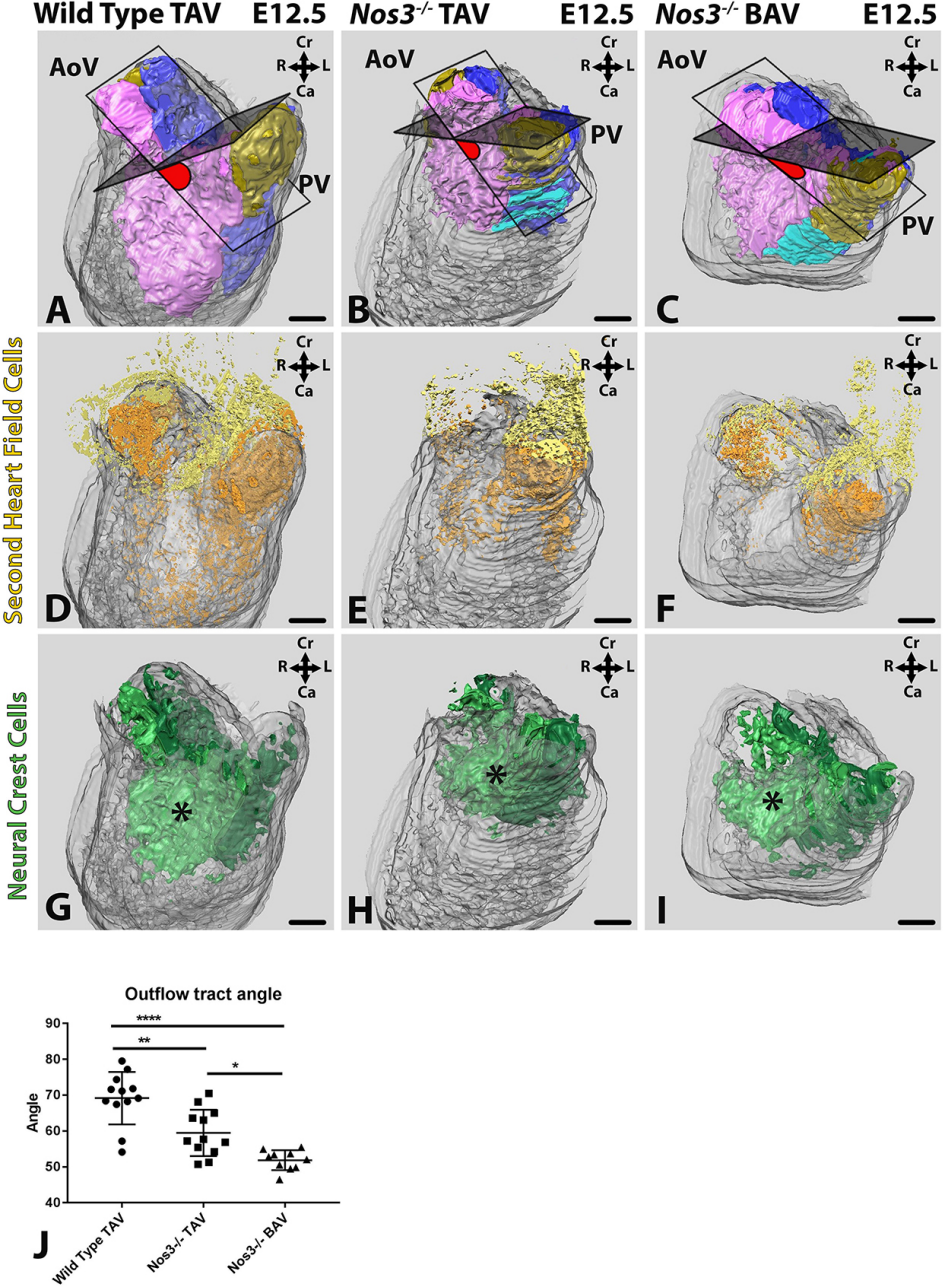
**Nos3 deficiency results in morphological outflow tract (OFT)** **defects in both tricuspid** **(TAV)** **and bicuspid** **(BAV)** **aortic valve mutants.** (A-I) 3D OFT reconstructions depicts myocardium (grey), non-coronary leaflet (gold), transitional region (cyan), parietal cushion (purple) and septal cushion (blue) of wild-type TAV (A), *Nos3^−/−^* TAV (B) and *Nos3^−/−^* BAV (C) embryos at E12.5. Protractor (red) of aortic- and pulmonary-valve commissure planes showed smaller angulation in *Nos3^−/−^* TAV and *Nos3^−/−^* BAV when compared to TAV wild-type embryos. NKX2.5^+^/TM^−^ second heart field (SHF) cells migrated from outside the cushions (yellow) into the cushions (orange) (D). Both tricuspid (E) and bicuspid (F) *Nos3^−/−^* mutants showed SHF cell populations restricted to the cushion tissue. (G-I) In TAV wild-type embryos (G) neural-crest-derived cells contributed to the formation of the aortopulmonary (AP) septum in regions of condensed mesenchyme positioned centrally between the aortic and pulmonary orifices (indicated by an asterisk). AP septum development in *Nos3^−/−^* TAV (H) and *Nos3^−/−^* BAV (I) is located more proximal to the semilunar valves than in wild type, affecting annulus formation and the position of the aortic and pulmonary valves. (J) Morphometric measurements between the aortic valve and pulmonary valve revealed significant differences between tricuspid wild-type and *Nos3*^−/−^ TAV (*P*=0.0012), as well as between wild-type TAV and *Nos3*^−/−^ BAV (*P*<0.0001). Moreover, there were significant differences between *Nos3*^−/−^ TAV and *Nos3*^−/−^ BAV (*P*=0.0159). This indicates a strong relationship between OFT development and leaflet formation, where a small angle between the aortic and pulmonary valve indices with BAV. Data are mean±s.d. *P<0.05, **P<0.01 and ****P<0.0001 determined by one-way ANOVA. AoV, aortic valve; PV, pulmonary valve; R, right; L, left; Cr, cranial; Ca, caudal. Colour coding: myocardium (transparent grey), parietal cushion (purple), non-coronary leaflet (yellow), septal cushion (light blue), pulmonary artery (dark blue). Nuclear DAPI staining: blue. Scale bars: 100 µm.

The aortic and pulmonary valve developed at an angular offset within the OFT of tricuspid wild-type embryos (Fig. 5A). NKX2.5^+^/TM^−^ SHF cells (Fig. 5D) and neural-crest-derived cells (Fig. 5G) were located deep within the OFT cushions in wild-type embryos. These neural-crest-derived cells contributed to the formation of the AP septum located centrally between the aortic and pulmonary valves (asterisk, Fig. 5G). In the OFT of *Nos3^−/−^* embryos, NKX2.5^+^/TM^−^ SHF cells and neural-crest-derived cells were located more proximal to the semilunar valves in both *Nos3^−/−^* TAV (Fig. 5B,E,H) and *Nos3^−/−^* BAV (Fig. 5C,F,I) mice. *Nos3^−/−^* TAV embryos showed proper leaflet separation but slight changes in the position of the AP septum were observed (asterisk, Fig. 5B,E,H). These effects were more pronounced in BAV *Nos3^−/−^* embryos, where the aortic valve was oriented more dorsally with regard to the pulmonary valve and changes in position of the AP septum were exacerbated (Fig. 5C,F,I).

Measurement of the position of the aortic valve in relation to that of the pulmonary valve (protractor, Fig. 5A-C) revealed that the angle between the aortic and pulmonary valves was significantly smaller in both tricuspid *Nos3^−/−^* and bicuspid *Nos3^−/−^* when compared to wild-type mice (*P*<0.01 and *P*<0.0001, respectively) (Fig. 5J). Moreover, TAV *Nos3^−/−^* and BAV *Nos3^−/−^* embryos were also found to be significantly different (*P*<0.05), indicating that the underlying changes in cell distribution may also affect annulus formation in tricuspid *Nos3^−/−^* mice.

## DISCUSSION

Patients with a BAV are at an increased risk of developing aortopathy. The great vessels and the aortic valve have a similar developmental origin involving contributions of endothelial, neural crest and SHF lineages. Understanding the exact role of these early cell lineages during leaflet development and OFT formation may lead to more accurate patient risk stratification and improved treatment strategies.

In this study, the formation of the aortic valve in wild-type and *Nos3^−/−^* mouse embryos was studied to identify congenital cell lineage aberrations involved in the formation of bicuspid aortic valves. To the best of our knowledge, we describe for the first time that the NF leaflets of the aortic and pulmonary valves arise from a process of cushion separation of the parietal and septal cushions during development. This finding contradicts previous studies, which argued that the formation of the NF leaflets developed from separate intercalated cushions (Kramer, 1942; Qayyum et al., 2001; Anderson et al., 2003; Lin et al., 2012; Eley et al., 2018; Mifflin et al., 2018). The morphological events leading to semilunar valve formation is a challenging concept to represent in a 2D-plane due to complex 3D OFT remodelling. Interpretations based on 2D histological sections may result in an incomplete view of the morphological changes throughout development. Moreover, these rapid morphological changes require short intervals in between observations and 3D reconstructions for accurate representations of the complete cushions within the context of the cardiac OFT remodelling during embryogenesis. The process of leaflet separation requires formation of endothelial infolding within the cushion. Fernández et al. reported fusion of the NC and RC (R-N BAV) as the underlying mechanism for BAV in *Nos3^−/−^* mice (Fernández et al., 2009). However, the absence of a strand of opposed endothelial cells that runs from the lumen to the sinus wall in the parietal cushion shows that the developmental background of the R-N BAV is not the result of a fusion process between two OFT cushions, but rather the result of an incomplete or absent separation process of the RC and NC leaflets from the parietal cushion during early valve development. We therefore found no indications of endothelial opposition that could support the theory of direct fusion. Recently, Eley and colleagues linked the formation of BAV without a raphe to deficiencies in arterial valve cells from *Mef2c-AHF-Cre^+^/Tnnt2-Cre^+^* progenitors (Eley et al., 2018). Interestingly, changes in NKX2.5^+^/TM^−^ SHF cell distribution are also observed in the *Nos3^−/−^* model, emphasizing the importance of correct cell lineage distributions during valve formation. Moreover, the cellular dynamics of the NKX2.5^+^/TM^−^ SHF population during OFT development observed in this study suggest that these cells do not originate from the myocardial wall as proposed by Mifflin et al. (2018) but rather migrate from the extracardiac pool of SHF cells into the OFT cushions, in agreement with Eley et al. (2018). However, the 3D reconstructions developed in our study do not indicate the formation of a detached/isolated developing intercalated cushion as the transitional zone did not develop into the NC or NF leaflets of the aortic and pulmonary valve. The transitional zones did not experience cushion swelling by cellularization as observed in the parietal and septal cushions but remained a thin region of cardiac jelly connecting the parietal and septal cushions during development. The development of the NC and NF from the septal and parietal cushions is further supported by the observations of the NKX2.5^+^/TM^−^ SHF cells as a distinct group within the parietal and septal cushion that eventually develop into the NC and NF leaflets of the aortic and pulmonary valve, respectively.

The asymmetric contribution of the NKX2.5^+^/TM^−^ population to the aortic and pulmonary poles of the OFT has been described as the pulmonary push concept (Scherptong et al., 2012). The greater number of contributing SHF cells to the pulmonary pole of the OFT in relation to that of the aortic pole might explain the susceptibility of *Nos3^−/−^* embryos to develop BAV as opposed to bicuspid pulmonary valve. The difference in cellular distribution within the pulmonary valve could possibly not reflect that of the aortic valve and could therefore be affected differently by the *Nos3* mutation. This subtle difference in cell lineage distribution might underlie the rarity of a bicuspid pulmonary valve in both mice and humans.

Our observations of cushion invagination for valve formation has also been reported in the vertebrate zebrafish model, suggesting a conserved mechanism for leaflet separation (Scherz et al., 2008). Whether the process of leaflet separation is the result of active endothelial invasion or a result of passive geometric rearrangement, e.g. by outgrowth of the valvular leaflets, is yet to be determined.

NOS3 is a key mediator in the production of nitric oxide (NO) in endothelial cells and plays a role in regulating vascular tone through the L-arginine–NO pathway (Radomski and Moncada, 1993). Disruptions in the endothelial NO pathway have been associated with BAV in humans (Kotlarczyk et al., 2016). However, the role of NO in OFT formation has not been studied in great detail. Signalling pathway analysis in *Nos3^−/−^* mice identified abnormal EMT as a cause for BAV (Fernández et al., 2009; Liu et al., 2013). However, these studies did not perform lineage tracing experiments and primarily relied on indirect measurements of EMT. We used *Tie2Cre;mTmG* mice to genetically label the endothelial-derived cells and showed that the endocardial contribution to the developing leaflets was not affected in *Nos3^−/−^* animals. Interestingly, the *Nos3* mutation did impact cell lineage distribution of neural crest and SHF cells among the leaflets of the aortic valve. This suggest that NOS3 might have an active role in guiding cell migration during development. Both SHF and neural crest cells migrate over the aortic and pulmonary arteries, in close proximity to endothelial cells. There have been reports that interactions between endothelial-derived cells and neural crest cell lineages are required for correct leaflet development (Wu et al., 2011).

Neural crest cells have been reported to be essential for correct positioning of the developing outflow cushions and patterning of the arterial valve leaflets (Phillips et al., 2013). More recently, Aghajanian et al. (2017) showed that endothelial Pdgfrα is an important factor for coordinating neural crest cell migration. The importance of correct lineage distributions of SHF, endothelial and neural crest cells in BAV formation has also been suggested by other researchers (Jain et al., 2011). The findings presented here support the notion that misregulation of these cell lineages can result in a BAV in mice.

The angular differences in the aortic and pulmonary valve plane between wild-type, *Nos3^−/−^* TAV and *Nos3^−/−^* BAV mutants as a result of lineage defects are likely to alter normal flow patterns. There is wide support that hemodynamic aberrations contribute to the aortopathy observed in many cases of BAV (Piatti et al., 2017).

To examine the exact molecular pathways responsible for the signalling role of *Nos3* in influencing neural crest and SHF distribution in OFT development, further research is required. Interestingly, the advent of RNA-sequencing can shed light on the molecular mechanisms involved in cellular communication among early cell lineages.

### Conclusion

During OFT development, the parietal endocardial cushion gives rise to the RC and NC leaflets of the aortic valve as well as to the RF leaflet of the pulmonary valve. The septal cushion gives rise to the LC leaflet of the aortic valve as well as the LF and NF leaflets of the pulmonary valve. This asymmetric leaflet formation leads to non-uniform cell lineage distributions in the aortic valve. The non-coronary aortic leaflet is primarily populated by NKX2.5^+^/TM^−^ SHF cells, whereas neural-crest-derived cells primarily populate the RC and LC aortic leaflets. Endothelial-derived cell populations contribute to each leaflet equally. However, *Nos3^−/−^* embryos develop BAV due to defects in endothelial-linked separation of the parietal cushion into the NC and RC aortic leaflet accompanied by a different pattern of disposition of embryonic cell lineages. All *Nos3^−/−^* embryos show increased contributions of SHF cells to the NC and LC leaflets, while a reduction of neural crest cells is observed in the RC leaflet. Moreover, embryonic lineage defects involved in valve formation result in morphometric changes of the OFT leading to aberrant positioning of the aortic and pulmonary valve in both tricuspid and bicuspid *Nos3^−/−^* embryos, although it is more pronounced in the latter. These valve alignment anomalies are most probably the result of aberrations in neural-crest-derived AP-septum formation in *Nos3^−/−^* embryos. These findings suggest that differences in early neural crest and SHF lineage distributions, as seen in BAV, are not limited solely to valve formation but impact complete cardiac OFT development.

## MATERIALS AND METHODS

### Embryonic material

Cardiac OFT and aortic leaflet development was studied in a series of developmental stages of *Nos3^−/−^* mice and compared to wild-type mice of the same age. The following mice have been used in this study: *Nos3^−/−^* B6.129P2-*Nos3*^*tm1Unc*^/J mice (purchased from Charles River Laboratories, Maastricht, The Netherlands); *Mef2cCre* mice were kindly provided by Dr QuinPing Feng (Ontario, Canada) (Verzi et al., 2005); B6.Cg-^*Tg(Wnt1-cre)2Sor*^/J (purchased from Jackson Laboratories, JAX stock #022501, Bar Harbor, USA); *Tie2Cre* mice were kindly provided by Dr Bernd Arnold (University of Heidelberg, Germany); and B6.129(Cg)-*Gt(ROSA)26Sor*^*tm4(ACTB-tdTomato,-EGFP)Luo*^/J (mT/mG) (purchased from Jackson Laboratories, JAX stock #007676). A breeding strategy was carried out to generate *Nos3^−/−^;Tie2Cre;mTmG*, *Nos3^−/^^−^;Wnt1Cre;mT/mG* and *Nos3^−/−^;Mef2cCre;mT/mG* transgenic mouse lines, which were used for lineage analysis. All mice were back-crossed to the Black6 background using C57BL/6JLumc mice [purchased from Leiden University Medical Center (LUMC), Leiden, The Netherlands].

Adult mice were bred overnight and embryonic age was determined according to the presence of a vaginal plug the following morning. Noon of the day that the plug was first observed was taken as embryonic day (E)0.5. Embryos were isolated through hysterectomy at E10.5-E16.5 following dissection in phosphate buffer solution, pH 7.4 (PBS). Tail biopsies were used for the isolation of genomic DNA. Genotyping was performed using polymerase chain reactions (PCR) for *Cre* and *Nos3* (Table S1). All mice were handled according to the Guide for Care and Use of Laboratory Animals, as published by the NIH, and the experiments were approved by the local (LUMC) animal welfare committee (dec14184).

### Immunohistochemistry

For histological examination, embryos were fixed in 4% paraformaldehyde (0.1 M, pH 7.4) for 24 h at 4°C. Subsequently, they were embedded in paraffin, sectioned serially (5 μm) and mounted on glass slides. Samples were deparaffinized with xylene followed by a series of graded ethanol steps for rehydration into PBS. Endogenous peroxidase activity was inhibited by exposure to 0.3% H_2_O_2_ for 20 min. Slides were subjected to microwave antigen retrieval in citric acid buffer (10 mM citric acid, 0.05% Tween 20, pH 6.0) for 12 min at 97°C. Sections were incubated with primary antibodies against NKX2.5, eGFP, TM and PECAM1. Primary antibodies were diluted in PBS–Tween-20 (PBST) with 1% bovine serum albumin (BSA, A8022; Sigma-Aldrich, St Louis, MO, USA) to prevent non-specific binding. Between subsequent incubation steps, all slides were rinsed twice in PBS followed by a single rinse in PBST. Tyramide signal amplification (TSA PLUS Biotin kit, NEL749A001KT, Perkin Elmer, Waltham, MA, USA) was used in NKX2.5 staining through addition of HRP-labelled antibodies followed by tyramide amplification according to the TSA PLUS Biotin kit manual. Primary antibodies were visualized by incubation with fluorescently labelled secondary antibodies, diluted in PBST for 60 min. Detailed antibody descriptions can be found in Table S2. DAPI (D3571, 1/1000; Life Technologies) was used as a nuclear stain and the slides were mounted with Prolong Gold (Life Technologies).

### Microscopic analyses and 3D reconstructions

3D reconstructions of the embryonic hearts were made with Amira software 6.3 (Template Graphics Software Inc., Houston, TX, USA) using a selection of *Nos3^−/−^;Tie2Cre;mTmG*, *Nos3^−/−^;Wnt1Cre;mTmG* and wild-type embryos, respectively, between E10.5 and E16.5 (Table S3). Tissue sections (5 µm) were collected from paraffin-embedded embryos and immunostaining was performed against NKX2.5, GFP, TM, PECAM1 and DAPI. Slides were scanned using the Pannoramic 250 Flash III slide scanner (3DHISTECH Ltd, Budapest, Hungary) and images of identical scale and exposure were exported using Histech Panoramic Viewer (3DHISTECH Ltd.). Subsequently, the photos were stacked and semi-automatically aligned in Amira. Relevant cardiac structures were labelled based on morphology and stains. Surface views were exported to PDF formats using the Adobe Acrobat 9.5 software package.

### Myocardial and endocardial cushion morphometry

The ventricular myocardium and the endocardial OFT cushion volumes were measured at ages E10.5-E13.5 by using the stereological method described by Gundersen et al. (1988). This method uses the random placement of evenly distributed points (grid) onto stained sections. Points within a tissue of interest are then counted on at least 10 sections, after which a reliable estimation of the real tissue volume can be made. The measured ventricular myocardium and endocardial cushion volumes were compared between wild-type (E10.5 *N*=3, E11.0 *N*=5, E11.5 *N*=5, E12.5 *N*=5) and *Nos3^−/−^* (E10.5 *N*=3, E11.0 *N*=4, E11.5 *N*=7, E12.5 *N*=6) embryos. A 100 mm^2^ grid was used to measure the ventricular myocardium (E10.5-E13.5) and endocardial cushion (E10.5-12.5) volumes. At older ages (E12.5), a 225 mm^2^ grid was used for the ventricular myocardium morphometry. The distance between two subsequently measured sections was 0.05 mm when measuring the myocardium and 0.025 mm for the endocardial cushion morphometry. An Olympus microscope was used with either a 40× or 100× magnification, depending on the size of the heart.

### Cell lineage analysis

Fluorescent images were collected using the Pannoramic 250 Flash III slide scanner (3DHISTECH Ltd.) or Leica Sp8 confocal microscope (Leica Microsystems, Buffalo Grove, IL, USA). Measurements were performed on aortic valves from transverse sections (5 µm) of wild-type C57BL/6JLumc (*N*=8), *Tie2Cre;mTmG* (*N*=5), *Wnt1Cre;mTmG* (*N*=4), *Nos3^−/−^* B6.129P2-*Nos3*^*tm1Unc*^/J (*N*=8), *Nos3^−/−^;Tie2Cre;mTmG* (*N*=4) and *Nos3^−/−^;Wnt1Cre;mTmG* (*N*=4). For each embryo, all sections containing the heart were imaged. Image analysis was performed using a macro designed in Fiji (Schindelin et al., 2012). The macro was designed to measure the nuclear volume of DAPI^+^ nuclei bound by cytoplasmic GFP as well as DAPI^+^ nuclei lacking cytoplasmic GFP for each individual leaflet. Briefly, collected images (8-bit) were contrast stretched until 0.4% saturation was reached, ensuring identical spectral intensity for all images. Leaflet perimeters of LC, RC and NC leaflets were manually selected, and DAPI^+^ nuclei that were located within cytoplasmic GFP were measured as lineage-specific nuclei. DAPI^+^ nuclei lacking cytoplasmic GFP were added to the total volume of DAPI^+^ nuclei of that individual leaflet. In the case of BAV, leaflet perimeters between RC and NC leaflets were defined following the shortest straight path from the observed endothelial infolding to the myocardial wall. Manual image threshold for GFP^+^ cytoplasm was set at a pixel intensity of 120 and DAPI^+^ thresholds were automatically detected using the ImageJ ‘default’ algorithm. Following complete leaflet measurement, volumetric calculations were processed in Excel 2007 (Microsoft, Redmond, WA, USA). Additional calculations as well as graphic and statistical analysis were performed in GraphPad Prism 7.0 for Windows (GraphPad Software, La Jolla, CA, USA).

### Angle measurement of aorta and pulmonary trunk

Angle measurements of the aorta and pulmonary trunk were performed on wild-type TAV (*N*=12), *Nos3^−/−^* TAV (*N*=12) and *Nos3^−/−^* BAV (*N*=10) mice. Serial slides stained with Resorcin Fuchsin were stacked and aligned with the Amira software version 6.3. Two planes were created through the top of the three commissure attachments of the aorta and pulmonary valves. In the case of BAV, the plane was set at the top of the two commissures and the plane perpendicular was determined by the smallest diameter of the aorta. The normal vector of both the aortic valve (dot product *V*_aorta_) and pulmonary valve (dot product *V*_pulm_) could then be determined. The angle between the aorta and pulmonary valves was calculated using the formula:

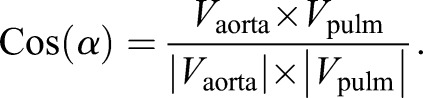


### Extracardial lineage analysis

Extracardial lineage analysis was performed on wild-type C57BL/6JLumc (*N*=3) and *Nos3^−/−^* B6.129P2-*Nos3*^*tm1Unc*^/J (*N*=3) embryos at stage E11.5. Immunostainings were performed using anti-NKX2.5 and anti-AP2α antibodies to visualize SHF and neural crest cells, respectively. Detailed antibody descriptions can be found in Table S2. 3D reconstructions and volumetric measurements of the extracardiac region adjacent to the cardiac OFT up to but not including the pharyngeal endoderm were made using Amira software 6.3 (Template Graphics Software Inc., Houston, TX, USA) and further processed in GraphPad Prism 7.0 for Windows (GraphPad Software).

### Statistical analysis

Results are represented as mean±s.d. of at least three independent experiments. Comparisons were made using unpaired two-tailed Student's *t*-test or one-way ANOVA test if data comparison involved more than two groups followed by Tukey's multiple comparisons test. Significance was assumed when *P*<0.05. Statistical analysis was performed in GraphPad Prism 7.0 for Windows (GraphPad Software).

## Supplementary Material

Supplementary information
