# Supplementary material for: Bicuspid aortic valve formation: Nos3 mutation leads to abnormal lineage patterning of neural crest cells and the second heart field
Source: Dis Model Mech. 2018 Oct 19;11(10):dmm034637. doi: 10.1242/dmm.034637 (PMC6215433; doi:10.1242/dmm.034637)
Supplement: Supplementary information [file dmm-11-034637-s1.pdf]

Supplemental Material

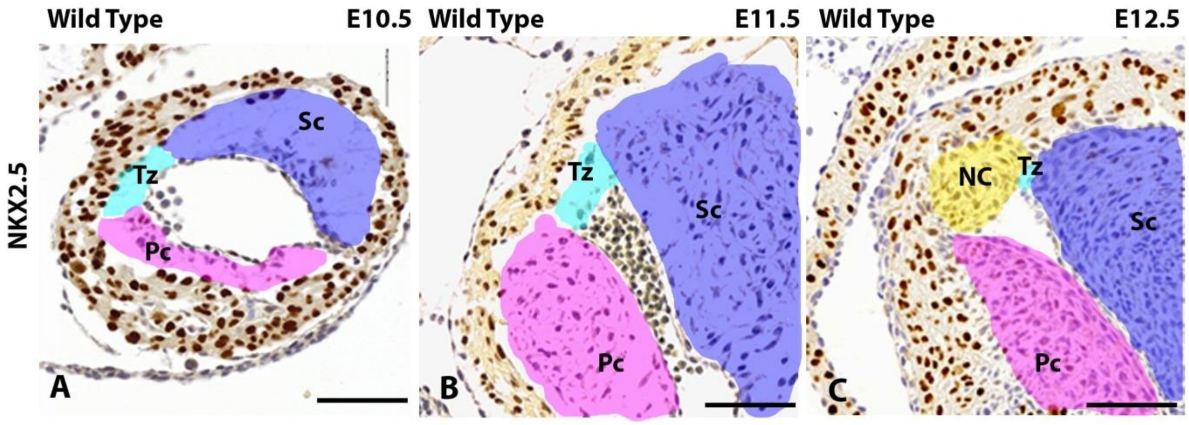

Figure S1.

Endocardial cushion development in wild type embryos

A: At E10,5 endocardial cells have migrated into the septal cushion (Sc) (blue) and parietal cushion (Pc) (purple). These cushions remained connected by a transitional zone (Tz) (cyan), a thin region of cardiac jelly sparsely populated by cells. B: The Pc and Sc enlarged at E11.5 whereas the transitional zone remained sparsely populated. C: At E12.5 the transitional zone was positioned at the site of the commissure between the left coronary leaflet (LC) and non-coronary leaflet (NC). The NC (yellow) was separated from the Pc. Myocardial cells were NKX2.5 positive (brown) . Scale bar is 50µm.

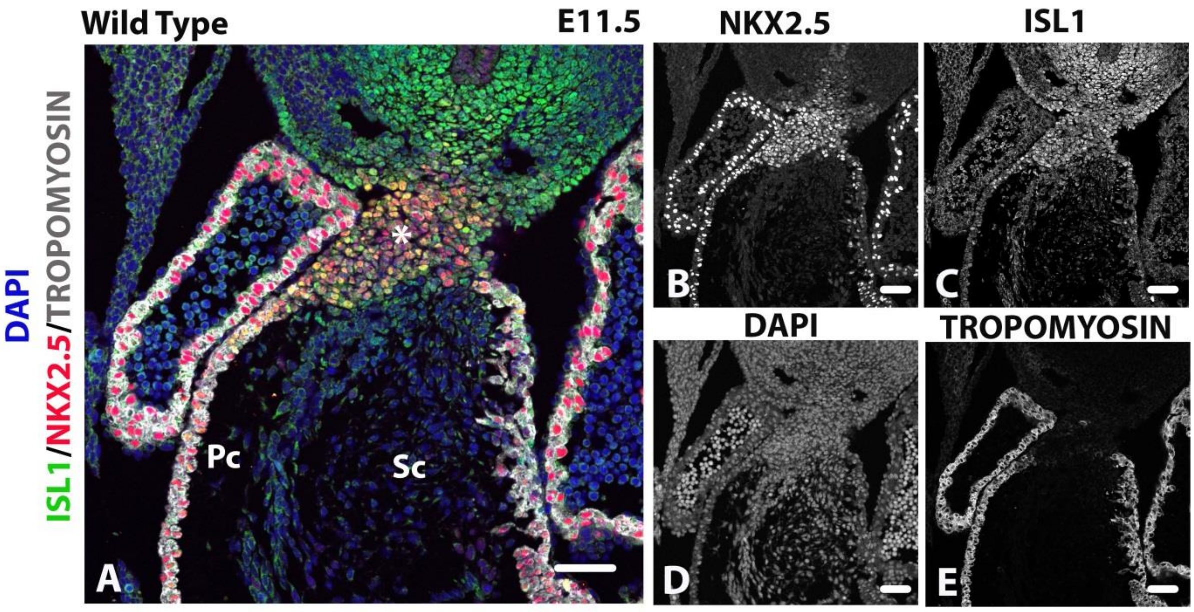

Figure S2.

NKX2.5⁺/TROPOMYOSIN⁻ cells represents ISL1⁺ second heart field cells contributing to the outflow tract.

A-E: Immuno-fluorescent images showing co-expression of NKX2.5 and ISL1 in second heart field cells contributing to the formation of the outflow tract in E11.5 wild type embryos. Colour scheme: Anti-NKX2.5 (red), Anti-ISL1 (green), Anti-TROPOMYOSIN (grey), DAPI was used as a nuclear staining (blue). Scale bar: 50µm.

Disease Models & Mechanisms • Supplementary information

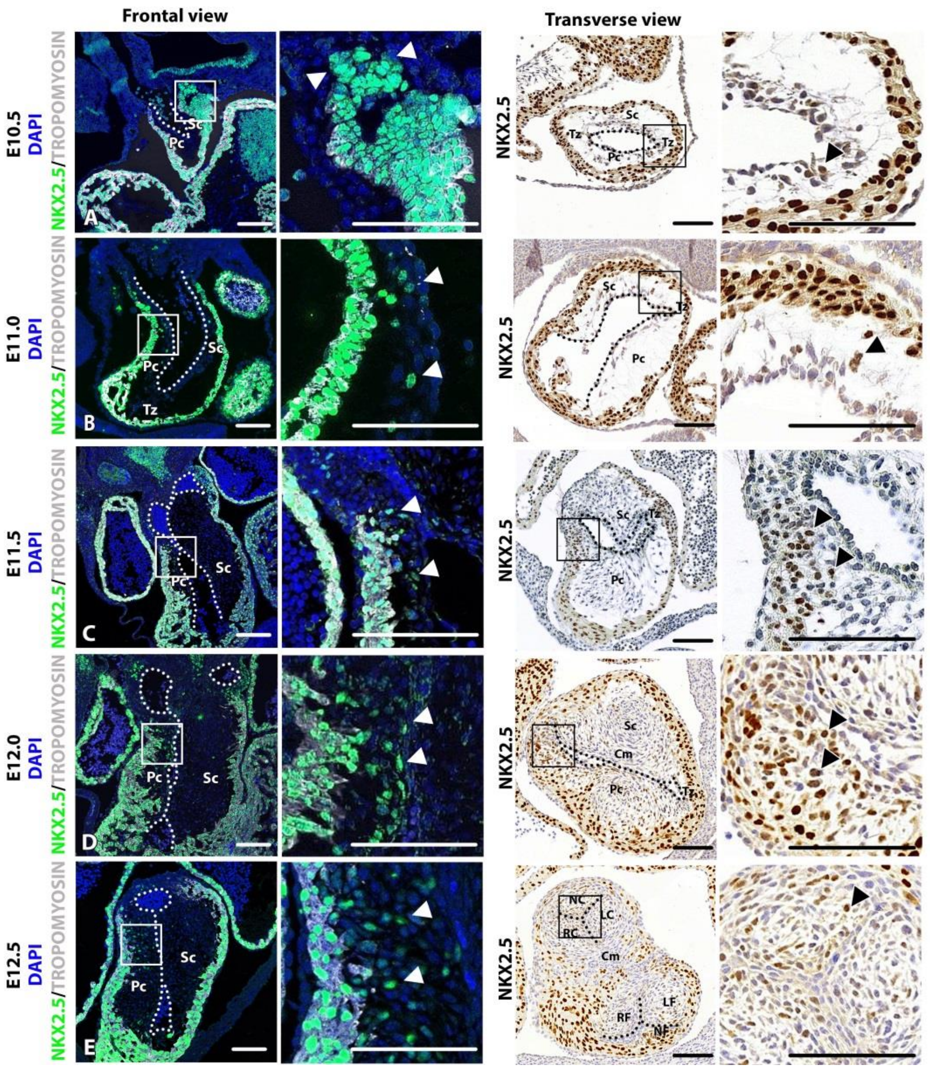

Figure S3.

Direct NKX2.5⁺/TROPOMYOSIN⁻ second heart field cells contribution to the aortic valve

A-E: NKX2.5⁺/TROPOMYOSIN⁻second heart field cells migrate into the cushions during development and contribute directly to the formation of the aortic leaflets. Colour scheme immunofluorescent images: Anti-NKX2.5 (green), Anti-TROPOMYOSIN (grey), Nuclei were stained with DAPI (blue). Colour scheme imunnohistological images: Anti-NKX2.5 (brown), nuclei are counterstained using hematoxine eosine (HE). Scale bar: 50µm.

Disease Models & Mechanisms • Supplementary information

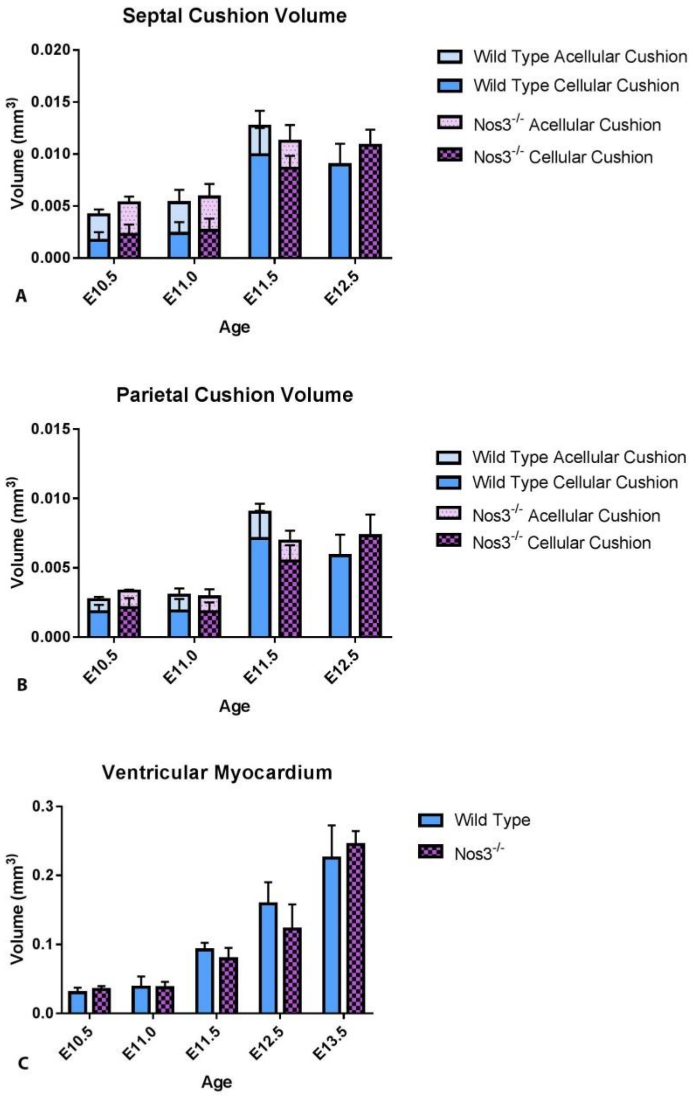

Figure S4.

Cushion volume is not affected in Nos3$^{-/-}$ embryos during stages E10.5 to E12.5 of embryonic development

A,B: Total cushion volume analysis indicated no differences ($p > 0.05$) in total volume of septal (A) and parietal (B) cushions between wild type and *Nos3*$^{-/-}$ embryos during development. Close examination also showed no difference in the cellular fractions of the septal and parietal cushions nor the acellular fractions between wild type and *Nos3*$^{-/-}$ embryos at stages E10.5 to E12.5. C: Myocardial volume analysis established that *Nos3*$^{-/-}$ embryos have an equal heart size compared to wild type embryos. Data are mean ± s.d. Analysis was performed using two tailed student T test.

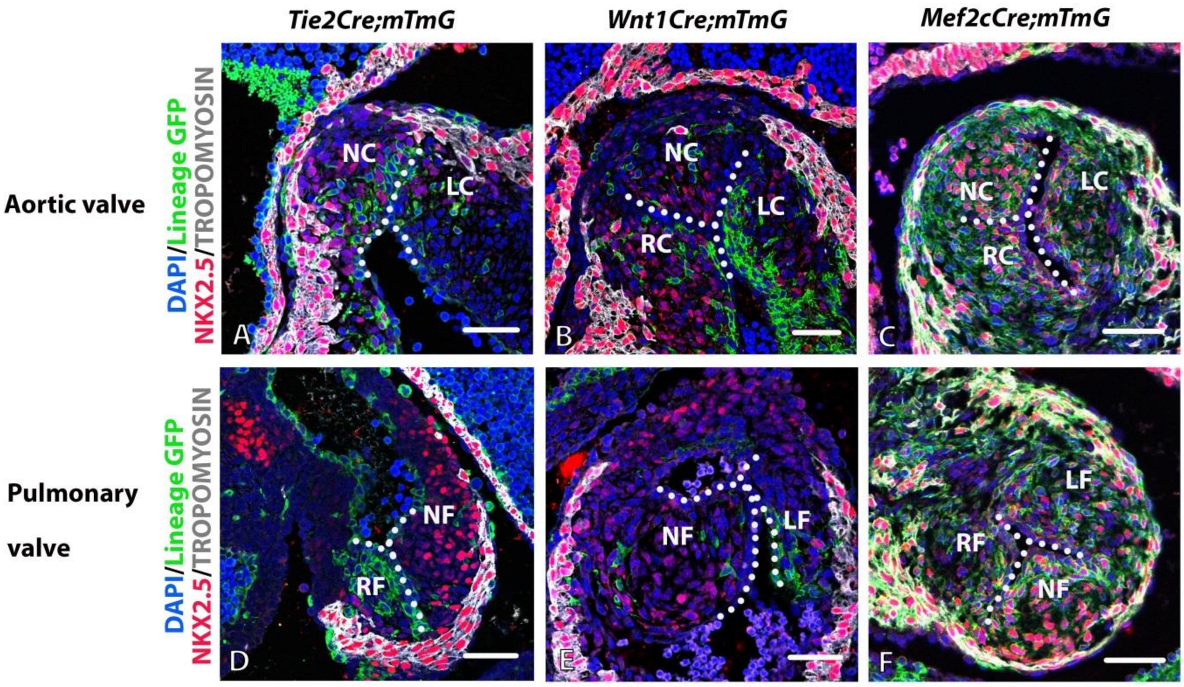

Figure S5.

Aortic leaflets have unique cell lineage distributions

A-F: Detection of NKX2.5 positive cells negative for TROPOMYOSIN indicated direct contribution of second heart field cells to the non-coronary aortic and non-facing pulmonary leaflet in wild type embryos at E12.5. NKX2.5^{+}/TROPOMYOSIN^{-} cells show no overlap with the *Tie2Cre* endothelial (A,D; green) or *Wnt1Cre* neural crest (B,E; green) lineages but do overlap with *Mef2cCre* second heart field derived cells (C,F; green). Dots indicate endothelial lining(white). Color scheme: TROPOMYOSIN (grey), DAPI (blue), NKX2.5 (red), Lineage-Cre derived GFP (green). Scale bar: 50µm

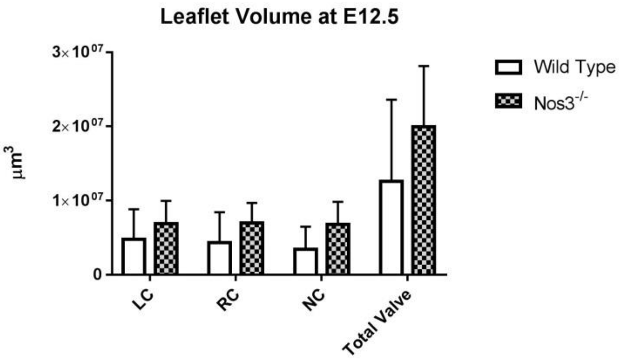

Figure S6.

Total leaflet volume is not affected in Nos3$^{-/-}$ embryos.

Volume analysis of aortic leaflets in the aortic valve shows equal volume in wild type and *Nos3$^{-/-}$* embryos.LC: left coronary leaflet, RC: right coronary leaflet, NC: non-coronary leaflet. Data are mean ± s.d. Analysis was performed using two tailed student T test.

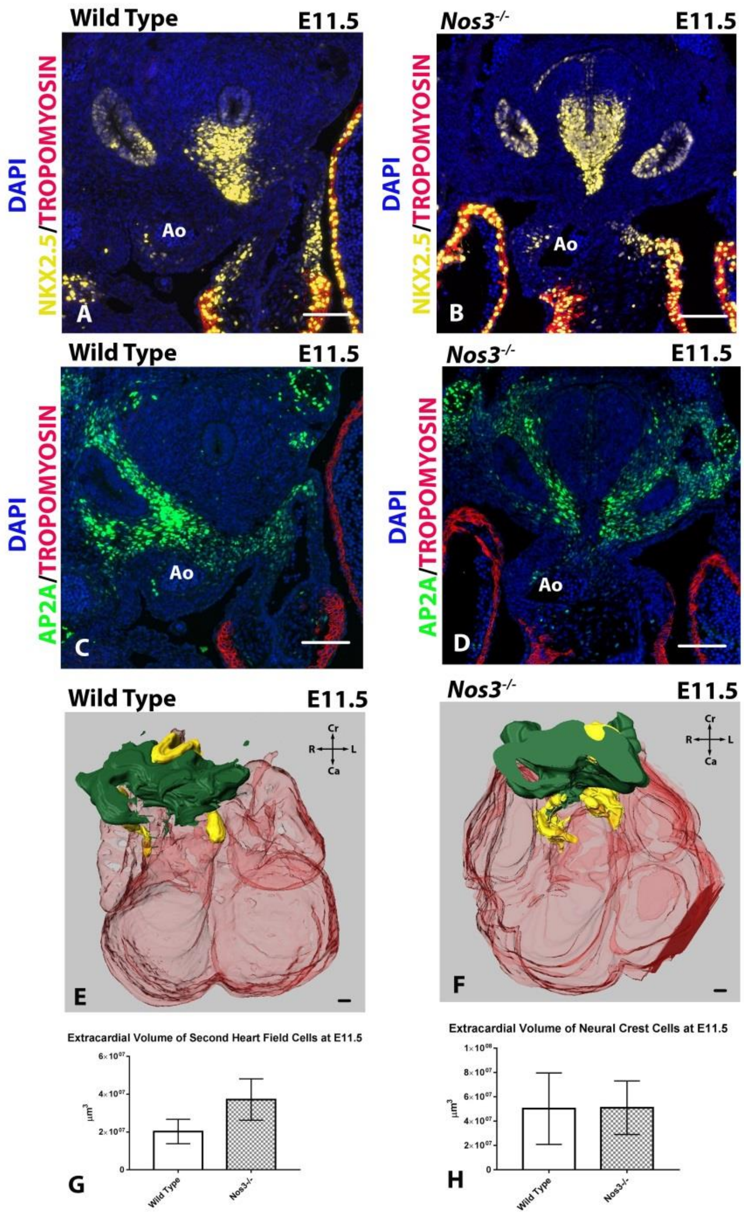

Figure S7.

Extracardial neural crest and second hear field population are not affected by Nos3 mutation.

A:D immuno-fluorescent images of extracardial NKX2.5^{+}/TROPOMYOSIN^{-} second heart field (SHF) (yellow) and AP2A neural crest cell (green) populations in wild type (A,C) and *Nos3*$^{-/-}$ (B,D) embryos at stage E11.5 when migration into the cushions has been initiated. E-F: 3D reconstruction of extracardial neural crest (green) and SHF populations (yellow) show similar localization in both wild type and *Nos3*$^{-/-}$ embryos. G-H: Volume analysis of extracardial populations shows no difference ($P > 0.05$) between SHF cells (G) and neural crest cells (H) between wild type and *Nos3*$^{-/-}$ at E11.5. Colour scheme: Anti-NKX2.5 (yellow), Anti-AP2A (Green), Anti-TROPOMYOSIN (red), Nuclei were stained with DAPI (blue). Data are mean ± s.d. Analysis was performed using two tailed student T test. Scale bars: 50µm

Fwd_Cre	ATG-GAT-TTC-CGT-CTC-TGG-TG
Rev_Cre	TTG-CCC-CTG-TTT-CAC-TAT-CC
Nos3_Mut_oIMR8963	AAT TCG CCA ATG ACA AGA CG
Nos3_WT_oIMR9357	AGG GGA ACA AGC CCA GTA GT
Nos3_Common_oIMR9358	CTT GTC CCC TAG GCA CCT CT
mTmG_WT_oIMR9021	CCG AAA ATC TGT GGG AAG TC
mTmG_Mut_22163	CGG GCC ATT TAC CGT AAG TTA T
mTmG_Common_oIMR9020	AAG GGA GCT GCA GTG GAG TA

Table S1. ***Primers used for genotyping***

**Primary Antibodies**	**Manufacturer**	**Dilutions**
Nkx2.5	Santa Cruz SC-8697	1/4000
eGFP	Abcam ab13970	1/500
Tropomyosin	Sigma-Aldrich Chemie T9283	1/500
PECAM1	Santa Cruz sc-1506-R	1/500
AP2α	GeneTex GTX62588	1/2000
**Secondary Antibodies**	**Manufacturer**	**Dilutions**
Horse Anti-Goat-Biotin	Vector labs BA-9500	1/200
HRP~Streptavidine - (PO)	Agilent P039701	1/200
Alexa Fluor488~Goat anti-Chicken IgY (H+L)	Thermo Scientific A-11039	1/200
Alexa Fluor 594~Donkey Anti-Mouse IgG (H+L)	Life technologies A-21203	1/200
Alexa Fluor 555~Donkey Anti-Rabbit IgG (H+L)	Life technologies A-31572	1/200
Cy5-Biotin	LifeSpan Biosciences ab6975	1/200

Table S2.

Antibodies used in this study

Disease Models & Mechanisms • Supplementary information

			Experimental Evaluation			
	Total embryos		Immuno-staining		3D Reconstructions	
Age	**Wild Type**	***Nos3*** $^{-/-}$	**Wild Type**	***Nos3*** $^{-/-}$	**Wild type**	***Nos3*** $^{-/-}$
**E10.5**	3	4	3	4	2	2
**E11.0**	7	6	7	6	1	0
**E11.5**	11	11	11	11	5	3
**E12.0**	4	2	4	2	1	0
**E12.5**	20	16	20	16	4	4
**E13.5**	14	11	14	11	2	1
**E14.5**	8	8	8	8	2	4
**E15.5**	3	8	3	8	0	0
**E16.5**	4	12	4	12	2	1

Table S3.

Number of embryos used in this study

**Age**	**BAV in WT**	**BAV in *Nos3*** $^{-/-}$	**Other cardiovascular anomalies in WT**	**Other cardiovascular anomalies in *Nos3*** $^{-/-}$
E12.5	0/20	4/16	0/20	0/16
E13.5	0/14	0/11	0/14	0/11
E14.5	0/8	2/8	0/8	0/8
E15.5	0/3	5/8	0/3	0/8
E16.5	0/4	4/12	0/4	0/12
**Mean**	**0/49 (0%)**	**15/55 (27.27%)**	**0/49 (0%)**	**0/55 (0%)**

Table S4.

Percentage of BAV found in wild type (WT) and Nos3 $^{-/-}$.

Disease Models & Mechanisms • Supplementary information
